# Vanishing Bone As the Presenting Feature of Multiple Myeloma

**DOI:** 10.7759/cureus.13861

**Published:** 2021-03-12

**Authors:** Nithisha Thatikonda, Uzma Rehman, Amol Gulkari

**Affiliations:** 1 Department of General Medicine, National Cancer Institute, Nagpur, IND; 2 Department of Radiology, National Cancer Institute, Nagpur, IND

**Keywords:** myeloma, vanishing bone, gorham-stout disease

## Abstract

A 47-year-old female presented with a minimally displaced fracture of proximal one-third of the shaft of the humerus that she suffered two months before and managed conservatively with a Plaster of Paris (POP) slab. An X-ray performed after six weeks showed complete vanishing of the proximal third portion of humerus due to massive osteolysis and generalized osteopenia. On examination, there was a soft tissue swelling over the proximal part of the left arm with painful and limited range of motion of the left shoulder. Biopsy of the lesion revealed neoplastic proliferation of plasmacytoid cells with binucleation showing strong, diffuse immunoreactivity to CD-138 and MUM-1, and thus a diagnosis of multiple myeloma was made. Typically, bone lesions in multiple myeloma present as multiple, well-defined "punched out" osteolytic lesions rather than complete vanishing of bone. The purpose of this report is to increase the awareness of this presenting feature of multiple myeloma.

## Introduction

Multiple myeloma is a malignant neoplasm of plasma B cells with 80% of patients having radiological evidence of skeletal involvement at the time of diagnosis [1-4]. Typically, bone lesions in multiple myeloma present as well-defined, multiple "punched out" osteolytic lesions with diffuse osteopenia or a single discrete osteolytic lesion (plasmacytoma) [2,3]. We report a case of multiple myeloma that looked like vanishing bone syndrome i.e. Gorham-Stout disease (GSD) at the time of initial presentation.

## Case presentation

**Figure 1:**
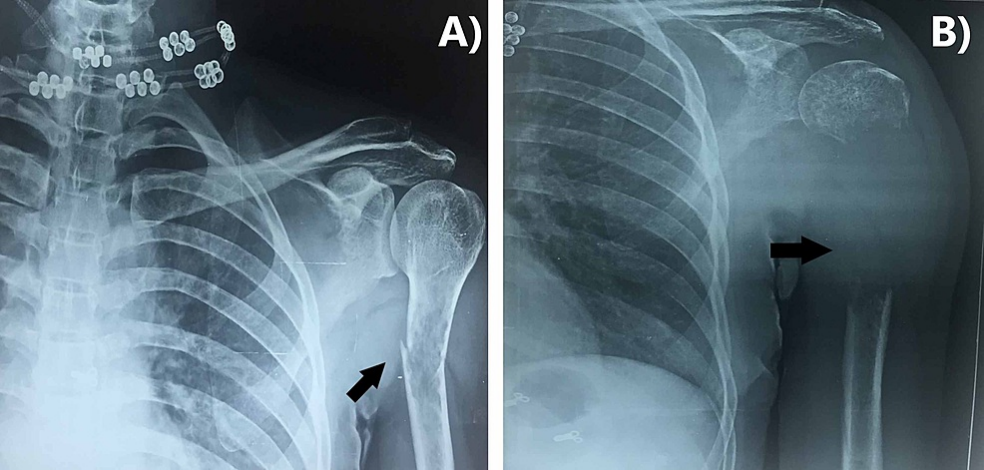
Plain X-rays showing fracture of proximal 1/3rd of the left humerus (A) and complete resorption of bone at the fracture site resulting in disappearance of the bone after six weeks (B).

**Figure 2:**
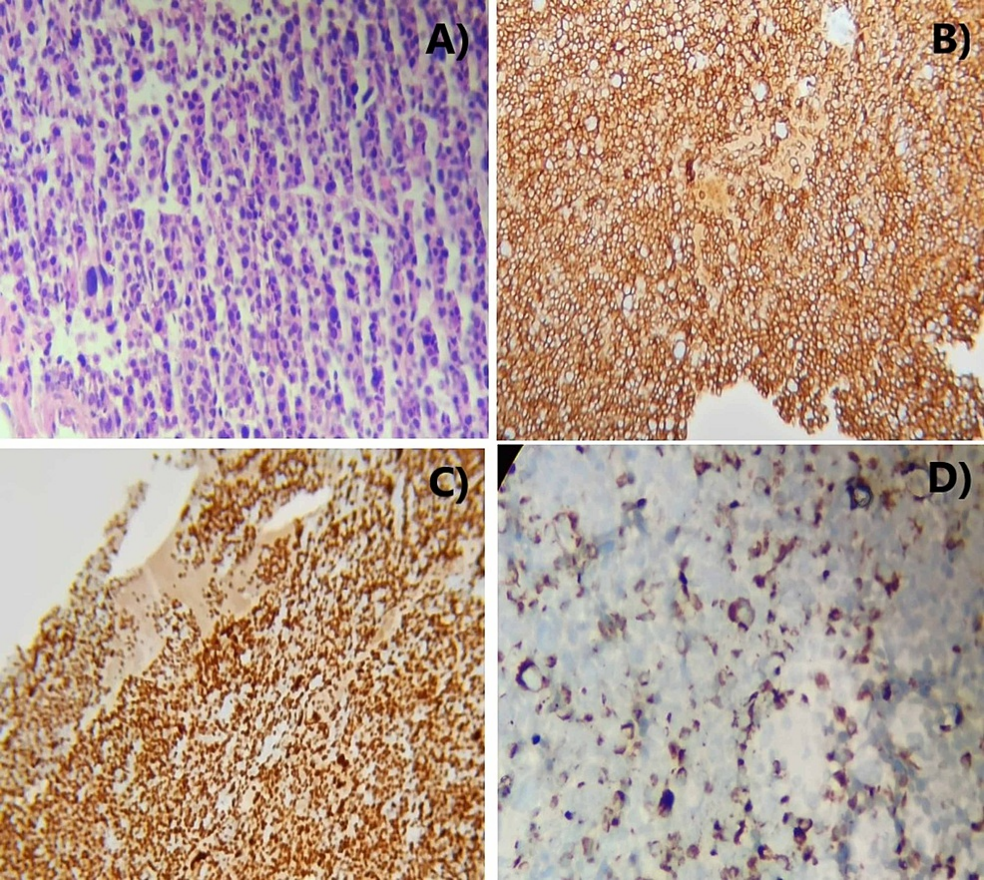
Biopsy of the lesion showing neoplastic proliferation of plasmacytoid cells with binucleation (A) showing strong, diffuse immunoreactivity to CD-138 (B), MUM-1 (C), and pan-CK (D).

An otherwise healthy 47-year-old woman was presented with a nonhealing fracture of the proximal one-third shaft of the left humerus, sustained two months prior after minor trauma. She was taken to the nearest hospital then, for left arm pain, where a plain radiograph of the left humerus showed a suspicious osteolytic lesion and minimally displaced transverse fracture at the proximal third shaft of the humerus (Figure 1A). Conservative management with a Plaster of Paris (POP) slab was done. An X-ray performed after a period of six weeks showed marked disappearance of the proximal third portion of the humerus due to massive osteolysis and generalized osteopenia (Figure 1B). On physical examination, the patient's left upper limb revealed soft tissue swelling over the proximal part of the left arm with a painful and limited range of motion of the left shoulder. Based on the rapid resorption of bone, vanishing bone syndrome (GSD), skeletal metastasis, hyperparathyroidism, and osteomyelitis were considered as differential diagnoses. Blood investigations showed hemoglobin of 7.1 g/dl with normal white blood cell count, serum calcium, and parathyroid hormone levels. Fine needle aspiration cytology and immunohistochemistry from biopsy were revealed neoplastic proliferation of malignant plasmacytoid cells with binucleation, as can be seen in Figure 2A, showing strong, diffuse immunoreactivity to CD-138, MUM-1, and pan-CK (Figures 2B-2D). Positron emission tomography (PET) scan revealed high fluorodeoxyglucose (FDG) avidity (SUVmax-7.12) in this lesion. Tiny osteopenic lesions were noted in the left scapula and T12 vertebra with mild FDG tracer uptake. Serum immunoelectrophoresis revealed monoclonal M band (102 g/dl) of immunoglobulin G (IgG) kappa variety and thus diagnosis of multiple myeloma was made and treatment was begun. At the time of writing this report, she received one cycle of bortezomib, cyclophosphamide, and dexamethasone with notable improvement in pain. Reconstructive surgery of the left humerus was planned after four cycles of chemotherapy.

## Discussion

Multiple myeloma is a malignant neoplasm of plasma B cells characterized by an overproduction of monoclonal immunoglobulins and marrow infiltration. At the time of diagnosis, radiological evidence of skeletal involvement is found in up to 80% of patients with multiple myeloma [1-4]. It can affect any part of the skeleton, preferably the spine (49%), skull (35%), pelvis (34%), ribs (33%), humeri (22%), femora (13%), and mandible (10%). These lesions usually present as single large osteolytic lesions most commonly in a vertebral body or the pelvis, diffuse osteopenia, or multiple, well-defined "punched out" lytic lesions [2,3]. However, in our case, the patient presented with a massive osteolytic lesion with gross destruction and disappearance of proximal third of humerus without any periosteal/surrounding sclerotic reaction. So, vanishing bone disease/GSD was considered one of the differentials initially.

GSD is a rare primary idiopathic osteolytic disorder characterized by proliferation of lymphatic and/or vascular structures of the bone suggestive of lymphangiomatosis and/or hemangiomatosis, resulting in destruction and resorption of the osseous matrix [5]. The syndrome can affect single or multiple bones with the humerus being one of the most commonly involved bones in most of the cases reported [5,6]. Clinical symptoms include pain, functional impairment, and swelling of the affected region often resulting in a pathological fracture in about 50% of the patients [7]. Our patient also presented with similar symptoms without any previous or family history of similar occurrences.

Few cases in the literature reported this vanishing bone presentation of multiple myeloma [3,4]. Our case closely resembled a case reported by Dolai TK et al, in which a plain X-ray of a 55-year-old female with pain and swelling of left arm revealed disappearance of midshaft of the left humerus which was akin to GSD. Biopsy of the lesion showed typical plasma cells having bi or tri nucleus and subsequently patient was diagnosed as having multiple myeloma [3]. Similarly, a 25-year-old HIV-positive female presented with a history of bilateral lower and upper limb pains with multiple soft tissue swellings for two years. Her plain radiographs showed multiple lytic expansile lesions with gross bony destruction and soft tissue swelling resembling GSD. Later, she was diagnosed with plasma cell myeloma based on histopathology findings [4].

The other differential to consider was osteolytic metastasis. However, the patient was not known to have any known malignancy at the time of presentation and her PET scan was also not suggestive of any other primary cancer site. Hyperparathyroidism and osteomyelitis were ruled out based on blood investigations.

## Conclusions

Bone lesions in multiple myeloma often present as a single discrete (plasmacytoma) or well-defined, multiple "punched out" osteolytic lesions with diffuse osteopenia. However, these lesions can also present as massive osteolytic lesions with gross destruction leading to complete bone resorption resulting in vanishing bone appearance. Recognizing this presenting feature of multiple myeloma is essential to increase the awareness on early detection of the disease based on initial X-ray findings.
